# Submicroscopic deletions of 11q24-25 in individuals without Jacobsen syndrome: re-examination of the critical region by high-resolution array-CGH

**DOI:** 10.1186/1755-8166-1-23

**Published:** 2008-11-11

**Authors:** Christine Tyson, Ying Qiao, Chansonette Harvard, Xudong Liu, Francois P Bernier, Barbara McGillivray, Sandra A Farrell, Laura Arbour, Albert E Chudley, Lorne Clarke, William Gibson, Sarah Dyack, Ross McLeod, Teresa Costa, Margot I VanAllen, Siu-li Yong, Gail E Graham, Patrick MacLeod, Millan S Patel, Jane Hurlburt, Jeanette JA Holden, Suzanne ME Lewis, Evica Rajcan-Separovic

**Affiliations:** 1 Department of Pathology and Laboratory Medicine and Child and Family Research Institute (CFRI), UBC, Vancouver, BC, Canada; 2 Department of Medical Genetics, UBC, Vancouver, BC, Canada; 3 Departments of Psychiatry and Physiology, Queens University, Kingston, ON, Canada; 4 Autism Research Program and Cytogenetics and DNA Research Laboratory, Ongwanada, Kingston, ON, Canada; 5 Department of Medical Genetics, University of Calgary, AB, Canada; 6 Medical Genetics, Credit Valley Hospital, Mississauga, Ontario, Canada; 7 Department of Medical Genetics, Victoria General Hospital, Victoria, BC, Canada; 8 Section of Genetics and Metabolism, Children's Hospital, Manitoba, Canada; 9 IWK Grace Health Centre, PO Box 3070, Halifax, Nova Scotia, Canada; 10 Medical Genetics, Centre Hospitalier Universitaire Sainte-Justine, Montréal, Québec, Canada; 11 Eastern Ontario Regional Genetics Program, Department of Genetics, Children's Hospital of Eastern Ontario, Ottawa, Canada

## Abstract

**Background:**

Jacobsen syndrome is a rare contiguous gene disorder that results from a terminal deletion of the long arm of chromosome 11. It is typically characterized by intellectual disability, a variety of physical anomalies and a distinctive facial appearance. The 11q deletion has traditionally been identified by routine chromosome analysis. Array-based comparative genomic hybridization (array-CGH) has offered new opportunities to identify and refine chromosomal abnormalities in regions known to be associated with clinical syndromes.

**Results:**

Using the 1 Mb BAC array (Spectral Genomics), we screened 70 chromosomally normal children with idiopathic intellectual disability (ID) and congenital abnormalities, and identified five cases with submicroscopic abnormalities believed to contribute to their phenotypes. Here, we provide detailed molecular cytogenetic descriptions and clinical presentation of two unrelated subjects with *de novo *submicroscopic deletions within chromosome bands 11q24-25. In subject 1 the chromosome rearrangement consisted of a 6.18 Mb deletion (from 128.25–134.43 Mb) and an adjacent 5.04 Mb duplication (from 123.15–128.19 Mb), while in subject 2, a 4.74 Mb interstitial deletion was found (from 124.29–129.03 Mb). Higher resolution array analysis (385 K Nimblegen) was used to refine all breakpoints. Deletions of the 11q24-25 region are known to be associated with Jacobsen syndrome (JBS: OMIM 147791). However, neither of the subjects had the typical features of JBS (trigonocephaly, platelet disorder, heart abnormalities). Both subjects had ID, dysmorphic features and additional phenotypic abnormalities: subject 1 had a kidney abnormality, bilateral preauricular pits, pectus excavatum, mild to moderate conductive hearing loss and behavioral concerns; subject 2 had macrocephaly, an abnormal MRI with delayed myelination, fifth finger shortening and squaring of all fingertips, and sensorineural hearing loss.

**Conclusion:**

Two individuals with ID who did not have the typical clinical features of Jacobsen syndrome were found to have deletions within the JBS region at 11q24-25. Their rearrangements facilitate the refinement of the JBS critical region and suggest that a) deletion of at least 3 of the 4 platelet function critical genes (*ETS-1, FLI-1 *and *NFRKB *and *JAM3*) is necessary for thrombocytopenia; b) one of the critical regions for heart abnormalities (conotruncal heart defects) may lie within 129.03 – 130.6 Mb; c) deletions of *KCNJ1 *and *ADAMTS15 *may contribute to the renal anomalies in Jacobsen Syndrome; d) the critical region for MRI abnormalities involves a region from 124.6 – 129.03 Mb. Our results reiterate the benefits of array-CGH for description of new phenotype/genotype associations and refinement of previously established ones.

## Background

Array-based comparative genomic hybridization (array-CGH) technology has vastly improved the resolution of cytogenetic analysis, resulting in the detection of pathogenic genomic imbalances in 9–17% of individuals with normal chromosomes and undefined causes of intellectual disability (ID) [1,2]. It has led not only to the description of new microdeletion/microduplication syndromes (for review see Slavotinek, 2008 [3]) but has also facilitated a more accurate genotype-phenotype correlation in cases of well established syndromes [4,5].

Jacobsen syndrome (JBS: OMIM 147791) was one of the first recognised contiguous gene syndromes, resulting from a terminal deletion of the long arm of chromosome 11 [6]. The typical clinical findings include thrombocytopenia, developmental delay, congenital heart disease and short stature [7]. Dysmorphic features include hypertelorism, ptosis, down slanting palpebral fissures, broad nasal bridge with short nose, thin upper lip and low set, malformed ears. In most cases, the partial monosomy of 11q is the result of a simple terminal deletion of 11q, with breakpoints usually occurring at or distal to 11q23.3. In around 25% of cases the 11q23.3 breakpoint has been localized to the FRA11B fragile site in 11q23.3 [7,8]. In a number of these patients it was demonstrated that there was a CCG triplet repeat expansion and expression of this fragile site in one of the parents [9]. Several other JBS deletion breakpoints in the 11q23.3-qter region were also shown to localize to CCG repeat expansions [10]. Interstitial deletions of 11q23.3-qter are rarely reported [11], as are cases of Jacobsen syndrome where the partial 11q monosomy was due to an unbalanced translocation (for example, Zahn et al. [12]). Several studies have provided a genotype-phenotype correlation in an attempt to identify critical regions for the most common physical abnormalities seen in Jacobsen syndrome [7,8].

We report here the clinical and molecular cytogenetic findings in two subjects with submicroscopic rearrangements involving the 11q24-25 JBS critical region, detected while screening 70 children with idiopathic ID and physical anomalies, using a whole-genome 1 Mb BAC array. The recurrent 11q24-25 rearrangements and associated phenotypes which are seen in our two patients are compared to those found in patients with Jacobsen syndrome.

## Methods

### Subject recruitment

Two subjects with 11q rearrangements were identified amongst 70 children with idiopathic ID having i) normal karyotypes by routine cytogenetic testing at 500–550 band level resolution, ii) negative Fragile X testing by DNA analysis, iii) a phenotype score ≥ 3 on the checklist presented by DeVries *et al *[13]***and ***iv) both parents available for testing. Clinical geneticists across Canada recruited subjects for genomic screening according to the above criteria.

### G-banded Chromosome Analysis

Metaphase chromosomes were prepared from peripheral blood samples using standard methods. GTG banding was performed according to standard procedures.

### 1 Mb BAC Array-CGH

Array-CGH was performed as previously described using the Spectral Genomics 1 Mb array [14]. The images were analyzed using SPECTRALWARE BAC Array Analysis Software v.2.0 (Spectral Genomics, Houston, TX). The positions of BAC clones were established using the database of genomic variants , human genome build 35.

### FISH

Fluorescence *in situ *hybridization (FISH) was performed with BAC DNA clones (Spectral Genomics, Houston, TX) or commercially available pre-labeled BAC clones (The Centre for Applied Genomics, Toronto) to confirm any deletions or duplications found by 1 Mb array-CGH [14,15]. Slides were viewed on a Zeiss Axioplan 2 fluorescence microscope, and images captured using MacProbe software (Applied Imaging, Santa Clara, CA). For each probe, at least 10 metaphase cells were analyzed. Interphase nuclei (50–100) were screened blindly in the case of microduplications.

### High Resolution Array-CGH

High resolution whole genome analysis was performed to refine the boundaries of rearrangements found by 1 Mb array-CGH, and also offered a second independent means of confirming the result. High-resolution Nimblegen 385 K array-CGH was performed courtesy of Nimblegen.

## Results

### Subject 1 (Figure 1a)

**Figure 1:**
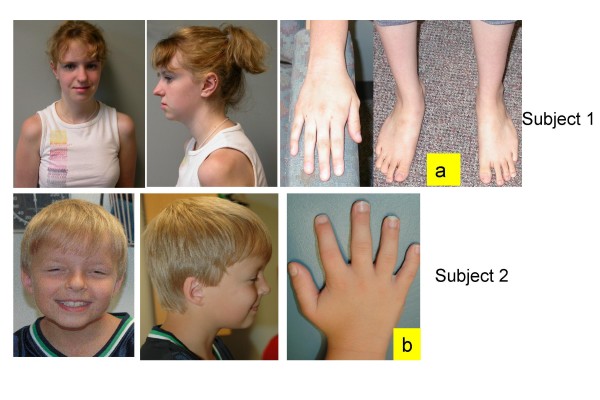
Facial features and physical abnormalities in subject 1 (a) and 2(b).

#### Clinical description

Subject 1 is the product of a second pregnancy to a non-consanguineous 32 year old mother and 34 year old father, both of northern European descent. She was born at 39 weeks gestation with a birth weight of 6 lb 11 oz. She was noted at birth to have a single umbilical artery, bilateral preauricular pits, bilateral simian creases, and ultrasound in early infancy showed an absent right kidney. Physical examination at 1 month of age showed an active alert female without dysmorphic facies; Occipital-frontal head circumference (OFC), length and weight were 50^th^, 75^th ^and 25–50^th ^percentiles, respectively. Neurological exam was normal. However, later assessment showed her developmental milestones were delayed: she walked at nearly 2 years, and began putting two words together at 2 1/2 years. Audiology testing demonstrated mild to moderate conductive hearing loss in the left ear, and a less severe right-sided loss. At 38 months, she was mildly unusual in appearance with a broad nasal root, widely set eyes, and preauricular pits. OFC was reduced to the 5^th ^percentile, relative to maintenance of height and weight approximating the 50^th ^percentiles. Her voice was noted to be monotone and nasal in quality. At age 6 1/2 years, concerns were noted regarding social skills, with difficulty making friends, an inability to concentrate and impulsiveness. She was noted to have difficulty with gross motor skills. Her systemic health was otherwise uncomplicated. At this time, OFC, height and weight were 25^th^, 25^th ^and 5^th ^percentiles, respectively. She had an unusual facies with a prominent forehead, receding hairline, and fine hair which grew slowly. She had a high nasal root, squarish nose, long philtrum, straight mouth and narrow upper vermilion border. She had a pectus excavatum, and was mildy hyperextensible. She was noted to be remarkably like her mother in her facial appearance. When examined at 9 years 1 month she had shown reasonable interval growth, lean habitus, mild pectus excavatum, and startling blue eyes. She continued to have behavioral concerns including impulsive behaviour and difficulty with strangers. Psychoeducational testing, including the WAIS III (Weschler Adult Intelligence Scales – Version III), at age 16 indicated a borderline verbal score, a performance score in the mild disability range and a full score within the mild ID range. When seen at age 17 years 10 months she was noted to have long facies, remarkable blue eyes, an overhanging nose with a bulbous tip, well marked philtrum, and protuberant lower lip. She had mildly short D4 and D5 metacarpals. OFC, height and weight were at the 2^nd^, 10^th ^and < 5^th ^percentiles, respectively. She had rapid speech that was difficult to understand. A renal ultrasound done at age 18 documented a left kidney of normal size and location, and a smaller ectopic right kidney. Other investigations included an ophthalmology exam, which noted intermittent exotropia with mild myopia. Karyotype, FISH for 22q11.2/13 microdeletions, Fragile X studies, urinary organic acids and serum amino acids were all normal.

#### Array CGH

**Figure 2:**
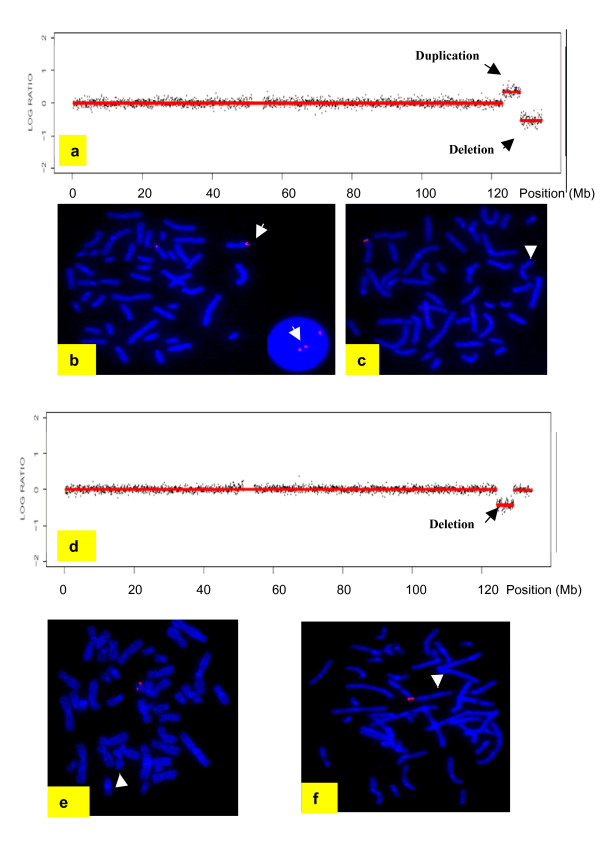
**11q24-25 rearrangements found in subjects 1 and 2**. (a) Nimblegen array (385 K) profiles of chromosome 11 showed a 5.04 Mb duplication and a 6.18 Mb deletion detected in subject 1. FISH confirmation of the duplication of clone RP11-50B3 (b) and deletion of clone RP11-206H19 (AP003775.3) (c) in subject 1. (d) Nimblegen array (385 K) profiles of chromosome 11 showed a 4.74 Mb deletion of chromosome 11q24.2-24.3 in subject 2. FISH confirmation of the deletion of clones RP11-50B3 and RP11-206H19 (AP003775.3) in subject 2 is shown in (e) and (f), respectively. Arrow indicates duplicated chromosome and arrowhead the deleted chromosomes.

A 5.04 Mb duplication (from 123.15–128.19 Mb) and adjacent 6.18 Mb deletion (from 128.25–134.43 Mb) at 11q24.2-25 were detected (Figure 2). FISH using BAC clones RP11-164A10 (dup), RP11-50B3 (dup), RP11-206H9 (del) and RP11-469N6 (del) mapping within the duplication and deletion regions confirmed the imbalances in the patient, and showed them to be *de novo *(Figure 2). The duplication probes showed a discordant (1:2) signal pattern in ~90% of interphase nuclei in subject 1 and < 20% nuclei in the parents, while the deletion probes showed a single signal in 100% of subject 1's interphase and metaphase cells (compared to < 20% nuclei in parents). The number of analyzed cells was 20–100.

### Subject 2 (Figure 1b)

#### Clinical description

This male child was initially seen in the Genetics Clinic at 8 months of age with developmental delay: specifically, the inability to sit unsupported or pull himself into sitting position, and macrocephaly (OFC > 98^th ^percentile). He was delivered at term following an uneventful pregnancy and his weight and OFC at that time were both at the 25^th ^percentile. At an early age he was noted to have a combination of motor and language delays as well as difficulties with socialization. MRI scans of the brain have shown evidence of abnormal white matter lesions, which were thought to be due to delayed myelination rather than a progressive leukodystrophy. At age 7 years the patient was re-assessed due to ongoing concerns regarding his development, as well his physical appearance. His medical history was unremarkable except for mild sensorineural hearing loss. He attended a regular classroom and was able to complete grade level work but required a full-time aide to help with some specific learning problems in reading as well as behavioral issues. His social interaction had improved significantly. His height, weight and OFC were 10–25^th^, 50^th ^and 98^th ^percentiles, respectively. He had relative macrocephaly with a prominent forehead (Figure 1b), a round face with a broad nose and slightly broad nasal tip with a well-grooved philtrum. His right ear was noted to be slightly smaller than the left and he had overfolding of the helices. His hands showed some 5^th ^finger shortening and squaring of the fingertips (Figure 1b). His physical appearance was not consistent with a diagnosable syndrome and a routine karytype as well as subtelomeric FISH analysis and Fragile X testing were normal. In reviewing the family history, his brother also has ID but with an otherwise very different, abnormal phenotype and an array detected *de novo *duplication of 19p13.3 (~3 Mb). There was no evidence of 11q24-25 abnormality in this brother. His parents and two older brothers are all healthy without any cognitive or learning disabilities and there were no reported paternity concerns.

#### Array CGH

A 4.74 Mb interstitial deletion was found at 11q24.2-24.3 (from 124.29–129.03 Mb) (Figure 2). FISH using BAC clones RP11-50B3 and RP11-206H9 confirmed the deletion and showed it to be *de novo *(Figure 2), as all 10 metaphases in subject 2 showed a hybridization signal on only one chromosome 11 homologue (compared to 10 parental metaphases which all showed signals on both chromosome 11 homologues).

## Discussion

We report the constellation of clinical and molecular cytogenetic features of two individuals with ID who have *de novo *submicroscopic rearrangements of chromosome 11q24-25. These two individuals are of particular interest since their deletions lie within the region that is commonly deleted in patients with Jacobsen syndrome [16]. Other than ID, neither of our patients exhibits the most characteristic features of Jacobsen syndrome, which typically include thrombocytopenia or pancytopenia, cranio-facial abnormalities (trigonocephaly) and cardiac defects [6]). As a result, it is possible to re-examine and refine the chromosomal regions responsible for the different phenotypes found in both our patients, and those with JBS.

### Paris-Trousseau syndrome (PTS)

**Figure 3:**
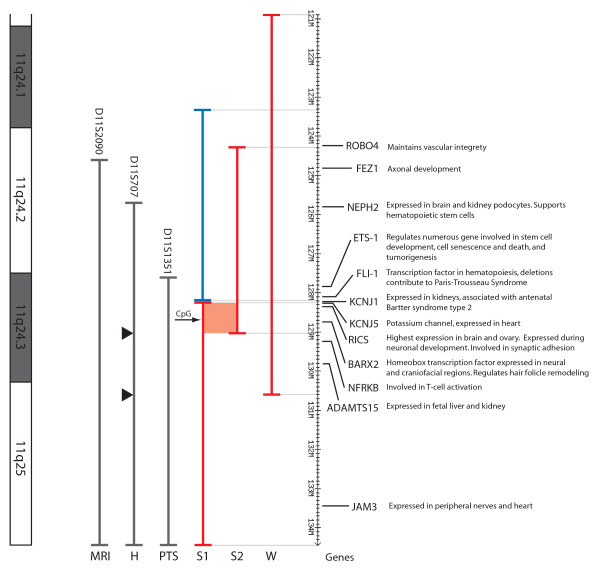
**11q24.1-25 region, showing the previously defined **[7]** critical regions for some of the common Jacobsen syndrome features, and the location of the rearrangements detected in the present subjects 1 and 2.** Chromosome bands are shown on the left, and the megabase (Mb) position on the right, with the positions and functions of some genes of interest. The patients' deletions are represented by red vertical lines, and the duplication of subject 1 by a blue vertical line. The 780 Kb region of deletion overlap between these two patients is shown by a red shaded box, and includes the CpG island 4929 (black arrow) that contains a (CCG)_6 _repeat. A third red vertical line labeled 'W' shows the size of the interstitial deletion reported by Wenger et al [11]. The critical regions [7] for MRI abnormalities (MRI), heart defects (H) and Paris-Trousseau syndrome (PTS) are shown by three vertical grey lines, with the proximal molecular marker indicated at the top of each line. A 1.57 Mb refined critical region for conotruncal heart defects is represented by two arrowheads (from the distal end of subject 2's deletion to the distal end of the deletion seen in the patient reported by Wenger et al [11].

This is the most common clinical finding (94%) in JBS patients, and is characterized by thrombocytopenia and platelet dysfunction. The critical region has been narrowed down to the 6.8 Mb terminal portion of 11q (distal to D11S1351, i.e. from 127.6 to 134.4 Mb) [7], and contains three genes associated with hematopoiesis (*ETS-1, FLI-1 *and *NFRKB*), and the *JAM3 *gene which is expressed in platelets [17] (Figure 3). Deletion of *FLI-1*, in particular, is thought to be at least partly responsible for the platelet/megakaryocyte defects observed in PTS patients [18]. The absence of PTS in subject 2, in association with the deletion of the *ETS-1 *and *FLI-1 *genes but the presence of the *NFRKB *and *JAM3 *genes, would suggest that the latter two genes, or other as yet unidentified genes, may be sufficient for normal platelet development and function (Figure 3). PTS also was absent in subject 1, whose terminal deletion includes *NFRKB *and *JAM3 *and who has duplication of *ETS-1 *and *FLI-1*. Therefore, it is likely that the platelet dysfunction and/or thrombocytopenia commonly observed in JBS might result from the combined effect of deletion of least three of these genes, and that deletion of only two of these genes is insufficient to cause the PTS phenotype. This is consistent with the finding of thrombocytopenia in a patient with an interstitial deletion of 11q24, which included *ETS-1, FLJ-1, NFRKB*, but excluded *JAM3 *(Wenger et al, [11]). In addition, as suggested by Penny et al [8], the genetic background of the individual might influence the expression PTS, since around 6% of patients with 11q terminal deletions do not have thrombocytopenia.

### Craniofacial abnormalities

Based on deletion mapping in JBS patients [8], a region influencing craniofacial development was speculated to map distal to D11S1351 (127.6 Mb), with *BARX2 *(128.75 Mb) as a potential candidate gene, since it is expressed in the neural and craniofacial structures during development [19]. *BARX2 *is further implicated here by its location within the small (780 Kb) area of deletion overlap for our two patients (Figure 3), both of whom display some of the facial dysmorphism (broad nasal root and a prominent forehead) that is seen in a significant proportion of JBS patients.

### Cardiac abnormalities

A variety of serious cardiac abnormalities is observed in individuals with JBS, and genes which influence cardiac development are believed to lie distal to D11S707 (125.8 Mb) [7] (Figure 3). Together, the deletions of subjects 1 and 2 span this entire critical region, yet neither individual has any heart anomaly. While the concurrent duplication in subject 1 may be able to somehow "compensate" for the loss of any heart-function related genes, the isolated interstitial deletion in subject 2 may be helpful to refine this critical region. When taken together with the subject reported by Wenger et al [11], a patient with conotruncal heart defects and a larger interstitial 11q24 deletion, one can see that one area critical to cardiac anomalies may reside in a 1.57 Mb region between 129.03 – 130.6 (i.e. from the distal end of subject 2's deletion to the distal end of Wenger's deletion, Figure 3). This small region contains several genes of interest; for example, polymorphisms in *SNX19 *have been reported to be associated with coronary disease [20], while *ADAMTS8 *is involved in regulating angiogenesis [21]. As suggested by Wenger et al [11], a second JBS cardiac critical region responsible for flow defects may be located distal to 130.6 Mb. This region contains *JAM3*, previously identified as a candidate gene for the JBS cardiac phenotype [22]. It is evident that the 11q JBS region is likely to contain a number of genes involved in cardiac function, which would account for the variety, as well as the presence/absence of heart defects observed in these patients. Indeed, genes outside the refined critical region (129.04 Mb to 11qter), such as *ROBO4 *(maintains vascular integrity) and *KCNJ5 *(highly expressed in heart) may also contribute to the cardiac defects that are observed in some JBS patients with larger deletions of 11q.

### Kidney abnormalities

Grossfeld et al [7] found structural defects of the kidney to occur in 8% of JBS patients. Two genes in 11q24.3, *KCNJ1 *and *ADAMTS15*, both of which are expressed in kidney, are potentially responsible for the renal anomalies found in subject 1. The *KCNJ1 *gene (at 128.2 Mb) lies within her duplication/deletion breakpoint, which might have disrupted the function of this gene, while *ADAMTS15 *lies within her terminal deletion region (Figure 3). In addition, the contribution of her 11q24.1-3 duplication, a region which includes *NEPH2 *(a gene which is known to be expressed in kidney podocytes and whose protein interacts with podocin), is unclear.

### MRI abnormalities

51% of JBS patients have abnormal brain imaging, with the critical region lying distal to 124.6 Mb (D11S2090) [7] (Figure 3). A region common to both this critical region and the deletion of subject 2, who has abnormal white matter lesions on MRI, is from 124.6 – 129.03 Mb. Potential candidate genes include *FEZ1*, predominantly expressed in the brain and involved in axonal outgrowth [23], and *RICS*, expressed during neural development and which may regulate dendritic spine morphology and strength [24].

In summary, although the large number of 11q24-25 genes that are imbalanced in subjects 1 and 2 (> 100) makes identification of genes responsible for specific phenotypic features challenging, the small 11q24-25 rearrangement sizes within the larger 11q JBS critical region are important contributors to understanding specific phenotype-genotype correlations. The high number of candidate genes located within the large, cytogenetically visible JBS deletions makes it likely that many of the typical features seen in JBS are due not just to monosomy of single genes, but to the cumulative effect of a combination of contiguous genes, or possible gene-gene interaction. This seems the most likely scenario, particularly when one considers the discrepancies that exist between phenotypes of some individuals, their deletion sizes, and the critical regions that are defined by the *majority *of patients with a particular phenotype. One should also keep in mind that the critical regions defined by Grossfeld *et al *[7] and Penny et al [8] were based on conventional karyotypes and a selection of DNA markers from the 11q terminal region. The use of whole genome array-CGH should facilitate a more accurate assessment of the types and sizes of chromosomal rearrangements in JBS.

The occurrence of a terminal deletion of 11q24.3 to 11qter, and duplication of 11q24.2-24.3 in subject 1 is a rare instance of a more complex rearrangement of 11q24-25 region. The duplicated region almost certainly will contribute to the patient's phenotype together with the concurrent deletion. Similarly, the effect of the deletion on the phenotypic expression of the 11q24.1-24.3 duplication should be taken into consideration when subject 1 is compared to other patients with duplications involving 11q24. In a review of 11q duplications, Delobel et al [25], showed that patients with interstitial duplications involving 11q23.3-24 do not have heart abnormalities, but commonly exhibit microcephaly, growth retardation, scoliosis and strabismus, which, except for the growth retardation, were not seen in our subject 1. The simultaneous occurrence of deletion and duplication in the 11q24-25 region in subject 1 may suggest the presence of low copy repeats (LCRs) which could lead to non-allelic homologous recombination, comparable to the 1 pter deletion/duplication event which can lead to 1 pter deletion syndrome [26]. The identity of such LCRs, however, remains unknown, since a comparison of segmental duplications within the 5 Mb region proximal to the duplication did not reveal significant homology. It is intriguing to consider the possibility that subject 1's terminal deletion/duplication could also be a result of a terminal deletion, followed by at least one breakage-fusion-bridge cycle, as was described for two cases with a terminal deletion/inverted duplication of 1 pter [27]. Detailed sequence analysis of the breakpoints in subject 1 would be required for the understanding of the molecular mechanisms leading to this rearrangement.

It would be interesting to perform whole genome screening of individuals with both typical and atypical Jacobsen syndrome, to test for the presence of complex rearrangements of the 11qter region and to rule out any additional rearrangements elsewhere in the genome. The results of such efforts would enhance our knowledge of the syndrome by allowing more accurate phenotype/genotype correlation.

## Conclusion

Our molecular cytogenetic and clinical findings in two subjects with submicroscopic 11q24-25 rearrangements facilitate the refinement of the JBS critical region and suggest that a) deletion of at least 3 of the 4 platelet function critical genes (*ETS-1, FLI-1 *and *NFRKB *and *JAM3*) is necessary for thrombocytopenia; b) one of the critical regions for heart abnormalities (conotruncal heart defects) may be within 129.03 – 130.6 Mb; c) deletions of *KCNJ1 *and *ADAMTS15 *may contribute to the renal anomalies in Jacobsen Syndrome d) the critical region for MRI abnormalities involves a region from 124.6 – 129.03 Mb. Our results reiterate the benefits of array-CGH for description of new phenotype/genotype associations and refinement of previously established ones.

## Abbreviations

ID: intellectual disability; JBS: Jacobsen syndrome; array-CGH: array-based comparative genomic hybridization; OFC: occipital-frontal head circumference; FISH: fluorescence *in situ *hybridization; WAIS lll: Weschler Adult Intelligence Scales – Version III; PTS: Paris-Trousseau syndrome; LCRs: low copy repeats

## Consent

Written informed consent was obtained from the patients for publication of this case report and accompanying images. A copy of the written consent is available for review by the Editor-in-Chief of this journal.

## Competing interests

The authors declare that they have no competing interests.

## Authors' contributions

CT and YQ made substantial contributions to conception and design, or acquisition of data, or analysis and interpretation of data, and have been involved in drafting the manuscript or revising it critically for important intellectual content. CH made substantial contributions to conception and design, or acquisition of data, and analysis and interpretation of data. XL made contributions to acquisition of data and analysis and interpretation of data. LA, FPB, BM, SAF, AEC, LC, WG, SD, RM, TC, MVA, SLY, GEG, PM, MSP and JH made substantial contributions to patient recruitment and acquisition of clinical data. JJAH, MESL and ESR made substantial contributions to conception and design, acquisition of data, or analysis and interpretation of data, and have been involved in drafting the manuscript or revising it critically for important intellectual content. ERS also gave final approval of the version to be published. All authors read and approved the final manuscript.
